# Force-activated separation devices: a preventive strategy for intravenous line disconnection in canine patients

**DOI:** 10.3389/fvets.2025.1547277

**Published:** 2025-03-14

**Authors:** Min-Jung Ko, Mu-Young Kim

**Affiliations:** Department of Veterinary Surgery, College of Veterinary Medicine, Konkuk University, Seoul, Republic of Korea

**Keywords:** force-activated separation device, line disconnection, line breakage, intravenous catheter, intravenous line, complications

## Abstract

Intravenous catheters are essential for administering medications and fluid therapy; however, complications such as line disconnection can occur, potentially leading to the discontinuation or delay of treatments. A force-activated separation device (FASD) can be installed between line components to help prevent these complications. Since the FASD has rarely been explored in veterinary settings, a survey of clinicians and clinical applications was conducted to evaluate the necessity, effectiveness, and considerations of this device. The survey revealed that approximately half of the respondents reported line disconnections as occurring “sometimes,” with patient-related causes being significantly more frequent than those caused by medical staff. Respondents noted that replacing a disconnected catheter typically required considerable time and at least two staff members. Despite this, over half of the respondents indicated that no preventive measures were in place. In clinical applications using the FASD, the overall disconnection rate across all patients was 44.3%, with the rate increasing with the patient’s body weight. Large dogs exhibited a disconnection rate of 80% (8 of 10), followed by medium dogs at 55.6% (10 of 18), and small dogs at 31% (13 of 42). Similar to the survey findings, patient-related causes were the most common for line disconnection. Improper separations occurred at a rate of 39.7%, most frequently between the device and the extension set. Line disconnection causes complications, requires manpower and time, and incurs costs. Therefore, preventive strategies are crucial from the perspectives of patients, owners, and clinicians. The FASD can be one of the strategies, particularly in large-breed dogs.

## 1 Introduction

Hospitalization is often required for veterinary patients with a wide range of clinical conditions. During hospitalization, securing vascular access is essential for administering medications and performing fluid therapy. Vascular access is available for a variety of purposes, from managing mild conditions to addressing emergent situations involving cardiovascular instability in veterinary medicine (1–5).

Although intravenous catheters (IVCs) offer significant advantages, they are also associated with complications such as extravasation, dislodgement, phlebitis, and occlusion, in both human and veterinary medicine (1, 4, 6). Among these, line breakage in canine patients is one of the most common complications, accounting for approximately 22% of total catheter-associated complications, as reported in previous studies (1, 5). Mechanical complications of intravenous catheters, including dislocation, occurred in 36 out of 39 catheters in another study (7). Line breakage can lead to the unexpected discontinuation or delay of medication or fluids, posing critical risks to emergent or unstable patients, and resulting in unnecessary delays and additional financial costs. These complications not only affect patient outcomes but also place significant strain on clinical resources, highlighting the importance of addressing IVC-related issues in veterinary care.

The SafeBreak Vascular Vet (Lineus Medical, Fayetteville, NC, United States), a force-activated separation device (FASD), is a breakaway device for peripheral IV lines. Installing between line components, the device separates when force-activated to prevent breakage-associated complications and fluid leakage, which would otherwise require IV replacement. *Simpson and Zersen* reported that complications, including line breakage, can be reduced by applying FASDs (8). According to their report, the complication rate was 8.9% in the group using the device, compared to 24.6% in the control group (8). Our study comprised two distinct phases. First, we conducted a survey of veterinarians to assess perceived frequencies and causes of line disconnection, along with associated clinical challenges. Second, a clinical analysis was performed to evaluate the actual incidence of line disconnection and improper device separation, identify contributing factors, and inform recommendations for patient selection and device optimization. Since previous studies have shown that the device is effective in reducing the frequency of adverse effects, this study specifically focused on line disconnection.

## 2 Method

### 2.1 Survey

The survey was approved by the Institutional Review Board (IRB) of Konkuk University. The survey was tested on a small group of participants before distribution. Online questionnaires were distributed to 58 veterinary clinicians across multiple institutions, including general and emergency practices. The survey included questions regarding the subjective frequency of intravenous (IV) line disconnection, its perceived causes, the time from the first detection of line disconnection to catheter replacement, the number of practitioners involved in catheter replacement, efforts to prevent line disconnection, and issues associated with line disconnection. The frequency of line disconnection was categorized as none, rarely, sometimes, often, and usually, and the frequency was assessed subjectively. Respondents were allowed to select multiple answers for certain questions, such as perceived causes, associated complications, time required to replace IVC, and the number of practitioners involved in catheter replacement. This approach was used because the causes and complications can vary, and the number of personnel and the time needed may differ depending on factors such as the patient’s size. The responses were collected and organized in Microsoft® Excel (Microsoft Corporation, Redmond, WA, United States) for subsequent analysis.

### 2.2 Clinical application

**Figure 1:**
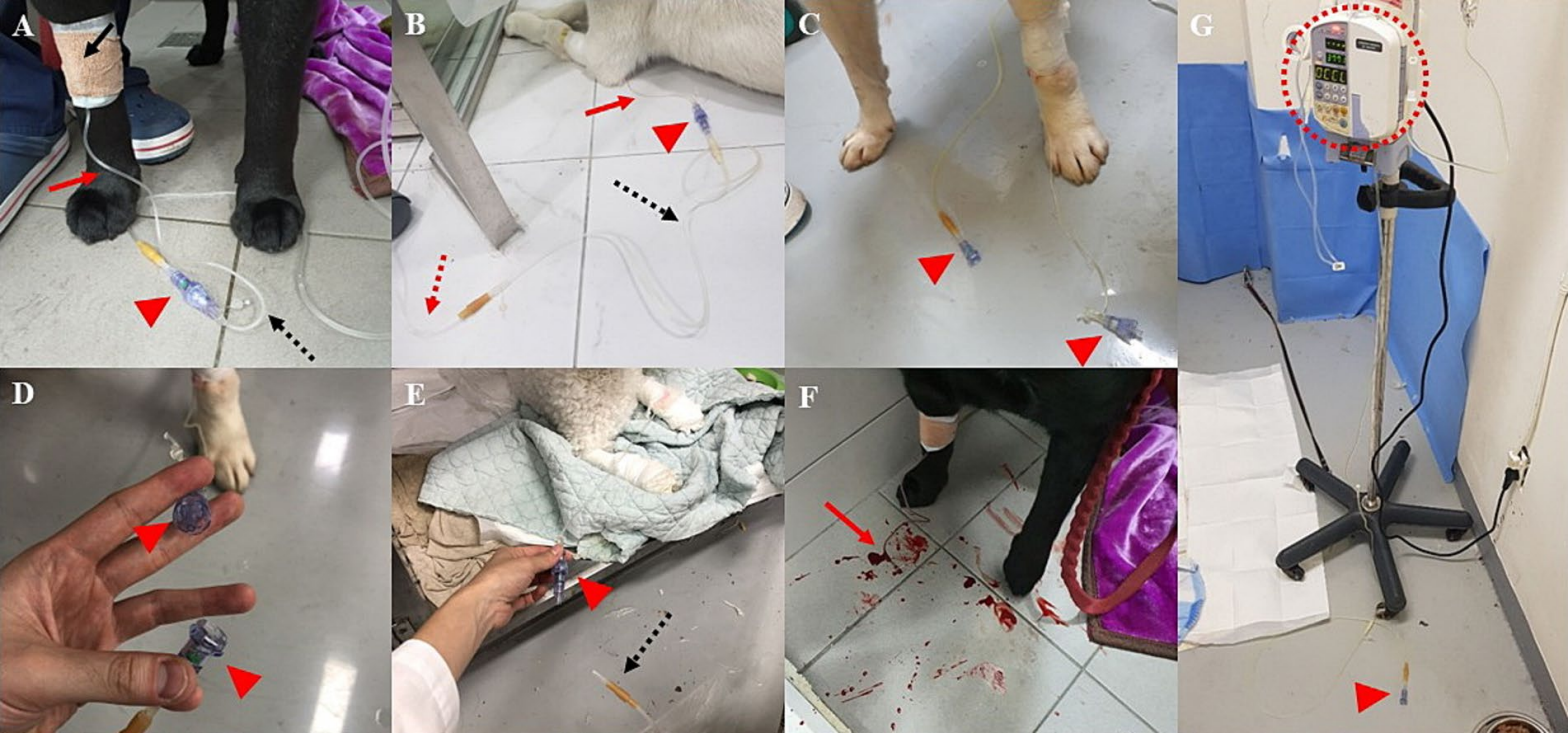
Clinical application of the SafeBreak device. In clinical application, the IVC line consists of an IVC connected with a heparin cap (not visible but the location is indicated by the black arrow), a butterfly needle (red arrow), the SafeBreak device (red arrowhead), an extension (black dotted arrow), and an infusion set (red dotted arrow). The components of the applied IVC line are identified in **(A,B)**. When the device is properly separated, it divides into two parts **(C,D)**. In some cases, improper location is separated. For example, improper separation between the device and extension occurs most frequently **(E)**. If the device is not applied and IV line disconnection occurs, associated complications such as unexpected bleeding **(F)** can occur. However, if the device is applied, a divided portion of the device closes preventing bleeding and fluid leakage by stopping the infusion pump **(G)**.

The clinical application was approved by the Institutional Animal Care and Use Committee (IACUC) of Konkuk University. This study involved 70 dogs hospitalized at the Konkuk University Veterinary Medical Teaching Hospital. All dogs had a hospitalization period of 3 to 5 days and required IVC placement for fluid therapy and medication administration. In each canine patient, the IVC line setup consisted of a catheter, heparin cap, butterfly needle, fluid extension, and fluid set, with an FASD installed between the butterfly needle and extension (Figure 1). The components were linked to each other by Luer slip, except for the FASD. The FASD setup did not follow the manufacturer’s recommendations and involved modifications, as the recommended setup for the FASD differed significantly from the setup used in clinical practice in South Korea. The Luer slip was used because it is more commonly used clinically due to its convenience and lower cost. Data collected included basic patient information such as age and body weight, as well as the occurrence of line disconnection during the hospitalization period, the site of disconnection, and the presumed cause. The data were organized in Microsoft® Excel (Microsoft Corporation, Redmond, WA, United States) and analyzed using statistical methods. A chi-square test or Fisher’s exact test was used to assess the associations between relevant factors and line disconnection or improper separation of the IV system. Data including age, body weight (kg), and status of the IVC line were collected. Age and weight were divided into three groups for statistical evaluation. Age was categorized as young (less than or equal to 5 years old), middle (6 to 10 years old), and old (over 10 years old). Body weight was categorized as small (less than or equal to 10 kg), medium (11 to 20 kg), and large (more than 20 kg). A commercial statistical software, SPSS (SPSS, Chicago, Illinois, United States), was used for statistical analysis.

## 3 Results

### 3.1 Survey

On the survey, the frequency of line disconnection, defined as a separation of any part of an IV line was reported as “sometimes” by 31 out of 58 (53.4%) respondents, whereas “rarely” was selected by 24 out of 58 (41.4%) respondents, indicating that these were the most common responses. The most frequently cited cause of line disconnection was active removal by patients (45 out of 57, 78.9%), followed by pulling on the IV line by patients (30 out of 57, 52.6%), and tube entanglement (28 out of 57, 49.1%). Line disconnection due to medical staff was reported at a significantly lower rate of 1.8% (1 out of 57).

The majority of respondents (28 out of 56, 50%) reported that the time from the first detection of line disconnection to catheter replacement took between 11 and 20 min, with 13 out of 56 (23.2%) respondents indicating that the time took between 21 and 30 min. Two medical staff members were most commonly required for catheter replacement (53 out of 57, 93%), with over half of respondents (30 out of 57, 52.6%) also reporting that three staff members were needed in some cases.

The primary issues associated with line disconnection were delays in medication administration (43 out of 58, 73.4%), patient stress due to reinsertion (36 out of 58, 62.1%), and reduced vascular access (29 out of 58, 50%). Preventive measures were not taken in 38 out of 58 (65.5%) cases. In response to taking measures to prevent line disconnection, 19 out of 58 (32.8%) participants reported taking passive actions subjectively, whereas 1 out of 58 (1.7%) reported taking proactive measures.

### 3.2 Clinical application

The overall IV line disconnection rate observed in this study was 44.3% (31 out of 70), and the rate with respect to relevant factors is summarized in Table 1. When analyzing the association between age and line disconnection, it was found that young patients (≤5 years) had the highest disconnection rate (17 out of 29, 58.6%), compared to middle-aged patients (6–10 years) with 8 out of 23 (34.8%), and older patients (≥11 years) with 6 out of 18 (33.3%). However, there was no significant association between age and line disconnection (*p* = 0.127).

**Table 1:** Factors and rate of line disconnection and improper separation of the device.

Factors		Total number	No disconnection	Disconnection	Improper separation
Age	Young	29	12	17	6
	Middle	23	15	8	5
	Old	18	12	6	1
Body weight	Small	42	29	13	8
	Medium	18	8	10	3
	Large	10	2	8	1

Regarding body weight, large dogs (≥20 kg) exhibited the highest disconnection rate (8 out of 10, 80%), followed by medium dogs (10 out of 18, 55.6%), and small dogs (13 out of 42, 31%). The rate of improper separation was 8 out of 13 (61.5%) in small dogs, 3 out of 10 (30%) in medium dogs, and 1 out of 8 (12.5%) in large dogs. A significant association between body weight and disconnection rate was observed (*p* = 0.010), with an increasing likelihood of line disconnection in heavier patients (*p* = 0.003). However, no significant correlation was found between body weight and improper separation (*p* = 0.173).

The most common cause of line disconnection was patient movement, accounting for 24 out of 31 (77.4%) cases, with improper separation occurring in 11 out of 24 (45.8%). The fault of medical staff was responsible for 7 out of 31 (22.6%) line disconnection cases, with a lower rate of improper separation of line disconnection at 1 out of 7 (14.3%) cases.

In terms of the location of disconnection, proper separation occurred at the device in 19 out of 31 (61.3%) cases (Table 2). Improper separation most frequently occurred between the device and extension set (9 out of 31, 29.0%), with no separation between the device and butterfly needle. Improper separation between the extension and infusion set accounted for 3 out of 31 (9.7%) cases.

**Table 2:** Rate of proper and improper separation of device and location of improper separation.

Location of disconnection		Number
Proper separation	At the device	19
Improper separation	Dislodged catheter from the vessel	0
	Between device and extension	9
	Between the device and butterfly needle	0
	Between extension and infusion set	3
Total		31

## 4 Discussion

According to the survey, the time required to replace an IVC line following line disconnection was generally 11–20 min, with most survey responses indicating that two staff members were needed for the procedure. A lack of available time or an insufficient number of free staff can lead to delays in the immediate replacement of the IVC line, and this may result in associated complications. Delay of medication, patient stress, and challenges in securing additional intravenous catheterization were the most frequently reported complications associated with line breakage. Despite this, 65.5% of respondents indicated taking no preventive measures, and only 1.7% actively took preventive measures such as strengthening the connection of line components by tape wrapping. This may be due to the lack of personnel available for immediate response, and it could also be because there are no adequate countermeasures in the current veterinary clinical environment. The other 32.8% of respondents adopted simpler preventive measures, such as paying more attention or applying an Elizabethan collar. Line disconnection causes various adverse effects. First, medical restraints required for IVC replacement can serve as uncontrollable stressors for veterinary patients according to various studies. They potentially lead to complications, including increased infection susceptibility, delayed wound healing, gastrointestinal distress, and subsequently elevated morbidity and mortality rates (9–11). Second, repeated attempts to access the IVC can result in vein collapse, ultimately causing failure in line replacement (12). Third, line disconnection can cause delayed medication. Medication errors including delayed medication are a patient safety concern in human medicine, leading to extended hospitalization periods, prolonged recovery times, increased pain, and higher mortality rates (13–15). Delayed medication is critical not only for patients with severe systemic conditions requiring insulin, anticoagulants, or other critical treatments but also for those with mild conditions that need simple antibiotics (14). Finally, additional costs are incurred. The additional costs were reported at a rate of 39.7%, which was lower than other complications on the survey. This may be attributed to the survey being conducted exclusively among veterinarians, rather than owners, for whom this may be a more significant concern. In human medicine, repeated attempts to access the IVC, as well as IVC-induced complications, are known to result in substantial costs (7, 12). Therefore, proactive efforts to prevent line disconnection are necessary from both the patient and owner perspectives.

The two most common responses to the questionnaire about the rate of line disconnection were “sometimes” and “rarely”, suggesting a low occurrence rate. However, it is important to note that this item was assessed subjectively, and recall bias may have influenced the accuracy of the participants’ responses. Upon reviewing the clinical data, the rate of device separation was found to be 44.3% and the proportion of improper separation relative to the total number of patients that received the device was 17% (12 out of 70). A previous study utilizing the same devices reported a device separation rate of 54%, with complications associated with improper disconnection of line components accounting for 6% (11 out of 180) (8). The differences in the location of the devices and the components of the IV line between the present study and the previous study may account for this discrepancy, potentially due to the variations in clinical settings across regions. Despite the variations in device location and line components, a proper separation rate was observed in 61.3% (19 out of 31) canine patients with line disconnection. Considering the proportion of proper separation in this study and the reduction in IVC complications in the previous study, these devices may function effectively in separating the IVC line, even in the presence of variations in the device location and line components.

Age and body weight were evaluated to determine their impact on line disconnection in clinical settings. While no significant associations were found between line disconnection and age, a significant linear relationship was observed between line disconnection and body weight. No correlation was found between body weight and improper separation of the device. Although the correlation between improper separation and body weight was not statistically significant, a decreasing trend in the likelihood of dislodgement was observed: 61.5% in small dogs, 30% in medium dogs, and 12.5% in large dogs. In the authors’ opinion, this suggests that a larger sample size might have revealed a statistically significant relationship. Consequently, it is possible that the FASDs could be more effective in dogs with higher body weight. The reason why small dogs show a higher rate of improper separation is not clear. However, unlike large dogs, where disconnection occurs due to strong force being applied temporarily while moving, small dogs tend to experience disconnections in various situations, such as when the line gets tangled, rather than just from momentary movements. It is likely that behavioral factors played a role.

The most frequent cause of line disconnection in clinical applications was patient interference (77.4%), as indicated by the survey. Specifically, the excessive patient movement resulted in line pulling, and spinning behavior often led to the entanglement of the line. Proper device separation was achieved in the majority of patients. The incidence of improper separation due to patient actions was higher than that caused by medical staff.

Related studies have shown that excessive movement and catheter line instability increase the likelihood of mechanical complications with IVCs (7, 16). Additionally, stress can lead to anxiety and heightened levels of activity and movement, which may further contribute to line disconnection (11, 17). In fact, one report indicated a higher rate of mechanical complications among veterinary patients who were not sedated (7). Benzodiazepines, dexmedetomidine, and trazodone can be administered as anti-anxiety medications for veterinary patients (11). Trazodone, in particular, has been shown to significantly reduce frenetic behaviors in hospitalized veterinary patients (9, 18–20). As factors such as separation from primary caregivers, unfamiliar environments, novel stimuli, noise, and disease also contribute to stress, addressing these aspects, alongside medication, may help reduce line disconnection rates by lowering patient stress and reducing excessive movement (10, 11).

The most common location of separation was between the device and the extension, followed by the connection between the extension and the infusion set. This suggests that the parts connected to the extension are the most mechanically vulnerable. Therefore, after applying the device, reinforcing these connections can help prevent improper separation and promote secure device fixation.

Several studies have indicated that longer hospitalization periods and higher APPLE scores (acute patient physiologic and laboratory evaluation), an indicator of disease severity, are associated with an increased frequency of IVC complications, including line breakage (5, 7). While uncollected factors in this study may have influenced catheter breakage and separation points, these were not evaluated, which represents a limitation of the current research.

The FASDs may also be beneficial for long-term intravenous placements, where prolonged use of IVCs increases the likelihood of mechanical issues such as line tension or accidental removal. Furthermore, FASDs could be explored in critical care scenarios, such as in patients requiring continuous fluid therapy. By reducing the risk of unintentional disconnections, these devices may contribute to improved patient outcomes and reduced treatment interruptions.

This study had several limitations. First, the small sample size may have influenced the statistical analysis, the interpretation, and the generalizability of the findings. Specifically, a trend toward improper separation was observed more frequently in patients with smaller body weights. With a larger sample size, a significant association might have been detected. Second, all patients in this study were equipped with the devices, and no control group without the FASDs was included for comparison. Third, potential biases, such as observer bias, could have influenced the outcomes. Finally, since all connections in the IV line system used Luer slip fittings except for the FASDs, the rate of proper separation may be lower than in systems using the Luer lock fittings. In South Korea, the majority of canine patients weigh less than 10 kg, and clinicians commonly prefer Luer slip systems to prevent the IV line from tangling around the patient’s neck or trunk. Luer slip tends to disconnect before twining occurs, which is a practical consideration in clinical settings. Therefore, our findings should be interpreted by considering these factors.

In conclusion, clinical data revealed a high occurrence of line disconnection, emphasizing the need for preventive measures. The use of a FASD could be one of the measures, particularly for large-breed dogs and high-risk patients, such as those prone to excessive movement, agitation, or self-trauma.

## Data Availability

The original contributions presented in the study are included in the article/supplementary material, further inquiries can be directed to the corresponding author.
